# Prediction of Coreceptor Tropism in HIV-1 Subtype C in Botswana

**DOI:** 10.3390/v15020403

**Published:** 2023-01-31

**Authors:** Kenanao Kotokwe, Sikhulile Moyo, Melissa Zahralban-Steele, Molly Pretorius Holme, Pinkie Melamu, Catherine Kegakilwe Koofhethile, Wonderful Tatenda Choga, Terence Mohammed, Tapiwa Nkhisang, Baitshepi Mokaleng, Dorcas Maruapula, Tsotlhe Ditlhako, Ontlametse Bareng, Patrick Mokgethi, Corretah Boleo, Joseph Makhema, Shahin Lockman, Max Essex, Manon Ragonnet-Cronin, Vlad Novitsky, Simani Gaseitsiwe, PANGEA Consortium

**Affiliations:** 1 Botswana Harvard AIDS Institute Partnership, Princess Marina Hospital, Gaborone, Botswana; 2 Department of Immunology and Infectious Diseases, Harvard T.H. Chan School of Public Health, Harvard University, Boston, MA 02115, USA; 3 Department of Ecology and Evolution, The University of Chicago, Chicago, IL 60637, USA

**Keywords:** CCR5, CXCR4, R5-tropic viruses, X4-tropic viruses, HIV-1C tropism

## Abstract

It remains unknown whether the C-C motif chemokine receptor type 5 (CCR5) coreceptor is still the predominant coreceptor used by Human Immunodeficiency Virus-1 (HIV-1) in Botswana, where the HIV-1 subtype C predominates. We sought to determine HIV-1C tropism in Botswana using genotypic tools, taking into account the effect of antiretroviral treatment (ART) and virologic suppression. HIV-1 gp120 V3 loop sequences from 5602 participants were analyzed for viral tropism using three coreceptor use predicting algorithms/tools: Geno2pheno, HIV-1C Web Position-Specific Score Matrices (WebPSSM) and the 11/25 charge rule. We then compared the demographic and clinical characteristics of people living with HIV (PLWH) harboring R5- versus X4-tropic viruses using χ^2^ and Wilcoxon rank sum tests for categorical and continuous data analysis, respectively. The three tools congruently predicted 64% of viruses as either R5-tropic or X4-tropic. Geno2pheno and the 11/25 charge rule had the highest concordance at 89%. We observed a significant difference in ART status between participants harboring X4- versus R5-tropic viruses. X4-tropic viruses were more frequent among PLWH receiving ART (χ^2^ test, *p* = 0.03). CCR5 is the predominant coreceptor used by HIV-1C strains circulating in Botswana, underlining the strong potential for CCR5 inhibitor use, even in PLWH with drug resistance. We suggest that the tools for coreceptor prediction should be used in combination.

## 1. Introduction

The envelope protein gp120 mediates Human Immunodeficiency Virus-1 (HIV-1) entry by interacting with the primary receptor (CD4) and a coreceptor (C-C motif chemokine receptor type 5 (CCR5) or C-X-C motif chemokine receptor 4 (CXCR4)) on target cells. Most HIV-1 viruses use the CCR5 coreceptor at the infection-initiation stage; these viruses are referred to as R5-tropic viruses [1,2]. Viruses using the CXCR4 coreceptor are referred to as X4-tropic viruses. In some cases, viruses use both the CCR5 and the CXCR4 coreceptors (i.e., dual-tropic viruses) [3]. In the early stage of HIV-1, viruses predominantly use CCR5 but can switch to CXCR4 at an advanced stage of the disease [4,5]. CXCR4 usage increases among patients on antiretroviral treatment (ART) [3,6,7]. A coreceptor switch to CXCR4 is associated with higher pathogenicity and has been reported in ~50% of HIV-1 subtype B infections but is rare and under-studied in subtype C infection [8,9].

Earlier studies of HIV-1C infection have shown a predominance of R5-tropic viruses, regardless of disease stage, suggesting that envelope characteristics may limit the emergence of X4-tropic viruses [10,11,12]. Other studies have identified dual-tropic viruses but no pure HIV-1C X4-tropic viruses [11,13]. More mutations may be required for a coreceptor usage switch in HIV-1C [8,14]. One study focused on 148 ART-naïve women with advanced stage of disease (CD4 cell counts below 200 cells/mm^3^) and found that ~15% of the women harbored HIV-1C dual-tropic viruses [13]. However, more recent studies have uncovered an increase in HIV-1C CXCR4 usage, which could reflect an evolving HIV-1C epidemic [3,15,16,17]. Among 24 treatment-experienced people living with HIV (PLWH) in Botswana in 2014, 33% had HIV-1C X4-tropic viruses based on Geno2pheno prediction [3].

Universal ART was rolled out in Botswana in June 2016 [18]. CXCR4 coreceptor use has implications for regimens that include CCR5 entry inhibitors, a class of anti-retroviral (ARV) drugs that inhibits viral entry by binding to cell surface CCR5 [19]. Maraviroc, an FDA-approved ARV, is only effective against R5-tropic viruses [19]. There is a need to monitor drug resistance and virus tropism, especially in patients failing treatment, to ensure effective administration of HIV treatment, particularly in a country with high rates of HIV and scaled-up HIV treatment, as is the case for Botswana. In Botswana, HIV prevalence was 20.3% as of 2018 [20] and Botswana has exceeded the UNAIDS 95-95-95 overall target by achieving 88% viral suppression, irrespective of previous diagnosis or treatment [21].

Phenotypic methods for determining HIV tropism are the gold standard but are expensive and laborious [22]. As an alternative, genotypic methods using bioinformatic tools that analyze viral genetic sequences are easily accessible, fast and inexpensive [23]. These tools predict viral tropism using the genetic sequence of the HIV-1 gp120 V3 loop. Earlier tools used the 11/25 charge rule by predicting X4-tropic viruses using the presence of charged basic amino acids in position 11 or 25 of the V3 loop [23]. More recent and computationally intensive tools model the 3D structure on the V3 loop and calculate the distance distribution between its functional groups [23]. These tools were developed for and have been applied mostly to HIV-1B [22]; thus, more research into the tropism of HIV-1C is required [22].

In the present analysis, we sought to predict the HIV-1 tropism of viruses among a large cohort of ART-naïve and treatment-experienced PLWH in Botswana.

## 2. Materials and Methods

### 2.1. Study Participants

Blood specimens were collected from a pair-matched community-randomized trial between 2013 and 2018, the Botswana Combination Prevention Project (BCPP). BCPP tested whether a strategy of treatment and interventions to prevent HIV infection would reduce the population-level cumulative HIV incidence [24,25]. A random sample of 20% of households in each community was selected. Consenting household residents aged 16–64 years who were Botswana citizens or spouses of citizens responded to a questionnaire and had blood drawn for HIV testing in the absence of documentation of positive HIV status. HIV testing was performed according to the Botswana national guidelines by using two rapid HIV tests in parallel. This study included both ART-naïve and ART-treated participants. ART status was confirmed through documentation of either clinical notes showing ART receipt, prescription or possession of pills [24]. Viral load testing was performed in all HIV-infected participants, irrespective of treatment status. Demographics and clinical characteristics collected included age, gender, ART status, virologic status and stage of infection (< or >1 year, as determined by longitudinal follow-up, where available).

The study received approval from the Botswana Health Research Development Committee (HPDME 13/18/1) and the US Centers for Disease Control and Prevention (Protocol 6475). The study is registered at ClinicalTrials.gov (NCT01965470). Participants who were aged 16–18 years provided written consent with parents or guardians providing written permission. All participants provided written informed consent.

### 2.2. Near-Full-Length HIV-1 Genotyping

A long-range HIV genotyping protocol was used to generate viral sequences, as described previously [26]. Minor modifications made to the protocol included reduced annealing temperature from 62 °C to 58 °C during amplification and using the first-round amplicon as a template for next-generation sequencing (NGS). The majority of participants were receiving ART and were virologically suppressed [27]; therefore, proviral DNA was used for amplification in 94% of participants. NGS was performed by the BioPolimers Facility at Harvard Medical School (https://genome.med.harvard.edu/, accessed on 12 June 2020) and through collaboration with the Phylogenetics And Networks for Generalised Epidemics in Africa (PANGEA) HIV consortium [28] (www.pangea-hiv.org, accessed on 25 January 2023) using Illumina platforms MiSeq and HiSeq [29]. In the present study, a single consensus sequence was generated and analyzed for each participant and majority-rule consensus was applied to assemble sequences generated by NGS. Of the 5602 HIV sequences, 5593 (99.8%) were generated by NGS, while the first 9 were generated by Sanger sequencing. Minor viral variants were not analyzed in this study.

### 2.3. HIV-1 Subtyping

Near-full-length HIV sequences were subtyped using online tools REGA HIV-1 Subtyping tool v.3 [30] and COMET v.2.3 [31]. Only HIV-1C sequences were included in this study (≥99% of the screened viral sequences in BCPP were HIV-1C). The V3-loop region (HXB2 nucleotide position 7110–7217) was extracted from the HIV-1C *env* gp120 region using the Los Alamos online gene cutter tool (http://www.hiv.lanl.gov/content/sequence/GENE_CUTTER/cutter.html, accessed on 10 March 2020).

### 2.4. Viral Tropism

Viral tropism was predicted using seven bioinformatic tools: Random forest, Boosted Decision trees, Neural Network, Support Vector Machine [32], Geno2pheno [coreceptor] v2.5, Web Position-Specific Score Matrices (WebPSSM) and the 11/25 charge rule. The first four methods are machine learning algorithms that use the V3 sequence and 3D protein structure to predict tropism (http://proteins.gmu.edu/automute, accessed on 28 August 2020) [32]. Geno2pheno, an online tool for coreceptor prediction [23] (https://coreceptor.geno2pheno.org accessed on 20 October 2021) was used with a 10% false-positive rate cut off (Geno2pheno_10%fpr_), as recommended by the European Consensus Group on clinical management for tropism testing for clinical use [33]. Geno2pheno classified the sequences into R5-tropic only or X4-tropic inclusive of the dual-tropic R5X4. Geno2pheno takes into account clinical information, CD4 cell counts and viral load, if available, in addition to merging the Support Vector Machine tool [23,34]. WebPSSM calculates scores from Position-Specific Score Matrices (PSSMs) (http://indra.mullins.microbiol.washington.edu/webpssm, accessed on 28 December 2021) and has been optimized for subtype C [35]. PSSM scores for subtype C were calculated as previously described [35]. Finally, we applied the 11/25 charge rule to determine tropism using an in-house script. X4-tropic viruses were identified as positively charged amino acids (K or R) present at position 11 and 25 of the V3 loop and/or subtracting the negatively charged amino acid (D or E) from the positively charged (net charge > +5). R5-tropic viruses are those that did not have positively charged amino acids (D or E) and/or had a net charge < +4. Most of the bioinformatics methods have been optimized for HIV-1B; however, Geno2pheno and WebPSSM have also been evaluated in HIV-1C, increasing their relevance to this study. For the concordance analysis between the concordant tools, we included the sequences where the tools agreeably predicted or failed to predict the viral tropism.

### 2.5. HIV-1 RNA Quantification

Plasma HIV-1 RNA levels were quantified using Abbott m2000sp/Abbott m2000rt (Wiesbaden, Germany). HIV-1 RNA above 40 copies/mL was considered a detectable viral load; lower viral loads were taken to indicate virologic suppression.

### 2.6. Statistical Analysis

We compared demographic (gender, age) and clinical (treatment status, viral load and stage of infection) characteristics for patients harboring CCR5 and CXCR4 viruses, and we excluded the sequences for which the viral tropism was not predicted by the tools. Age was categorized as 16–25, 26–35, 36–45, 46–55 and 56–64. Stage of infection was determined to be either recent (<1 year) or chronic (>1 year), where data were available. An infection was classified as recent if the participant had a recorded negative HIV test less than a year before their positive diagnosis at the beginning of the trial, or if the participant seroconverted during the trial with a recorded negative less than 1 year before that. Infections were classified as chronic if participants were HIV positive at enrolment and had recorded evidence of a positive HIV test > 1 year before the trial. Participants for whom stage of infection could not be determined were not included in this analysis.

For categorical variables, the χ^2^ test was used to compare groups, and the Wilcoxon rank sum was used for continuous variables. *p*-values < 0.05 were considered statistically significant. Data analysis was performed in STATA software 14 (Stata Corp., College Station, TX, USA) and R version 3.6.0, 2019 (Vienna, Austria).

## 3. Results

### 3.1. Genotyping and HIV Tropism Prediction

The V3 loop sequences were available for 5602 BCPP participants infected with HIV-1C. Coreceptor tropism was determined for the majority of the sequences (Table 1). Some methods, such as Random Forest, Support Vector Machine, Boosted Decision Tree and Neural Network, were sensitive to sequence length and could not process sequences shorter than 35 amino acids (437 failures). WebPSSM showed more tolerance for predicting tropism with sequences shorter than 35 amino acids (15 failures). The 11/25 charge rule performed successfully for all 5602 sequences. Overall, 16 sequences, which were not predicted by all seven tools, were included when combining the results from seven tools for concordance analysis. Viral load was not associated with success in determining viral tropism (*p* > 0.05).

The proportion of PLWH predicted to harbor R5-tropic viruses varied across the seven tools (Table 1): Boosted Decision trees classified 95.1% as R5; Random Forest, 94.2%; 11/25 rule, 93.2%; Support Vector Machine, 88.5%; Geno2pheno, 88.3%; Neural Network, 87.2%; and WebPSSM, 61.9%. All these tools classify dual-tropic viruses as X4-tropic viruses. The Support Vector Machine and Neural Network classified 11.5% and 12.8% of viruses as X4-tropic, respectively. Among all the tools, fewer than 15% of viruses were predicted to be X4-tropic, except for WebPSSM, which classified 38.1% of sequences as X4-tropic. Only three tools were selected for further analysis: Geno2pheno, WebPSSM and 11/25 charge rule. The Support Vector Machine tool was excluded because it is included in the Geno2pheno algorithms, while Neural Network, Boosted Decision trees and Random Forest were excluded due to their increased sensitivity to sequence length and could not process sequences shorter than 35 amino acids (437 failures). These methods predicted viral tropism in 5586 (99.7%) of analyzed HIV-1C sequences.

### 3.2. Concordance between Geno2pheno, WebPSSM and 11/25 Rule Tools

Concordance in determining viral tropism between the three methods was 63.9%, as shown in Table 2, including (*n* = 3367) R5-tropic and (*n* = 196) X4-tropic viruses. The concordance between the three tools included the sequences where the methods agreeably predicted or failed to predict the viral tropism. The highest concordance, 88.5%, was found between Geno2pheno and the 11/25 charge rule, while Geno2pheno and WebPSSM had the lowest concordance at 68.2%. We included a graphical representation (Figure S1 and Table S1 in Supplementary Data) showing the prediction of X4-tropic viruses across the three tools. There was no difference in tropism and the sampling date in the concordantly determined samples (*p* = 0.90) (Table S2 in Supplementary Data).

We then evaluated virus sequences sampled in Botswana cohorts from 1996 and from 2003, where HIV-1C tropism was predicted using phenotypic methods [13]. We predicted tropism genotypically using the same set of sequences by three tools, Geno2pheno, WebPSSM and the 11/25 charge rule. The 41 HIV-1C V3 sequences were phenotypically characterized and they are unique, as they were all from Botswana and represent the viruses in the early epidemic. Among 41 HIV-1C V3 sequences, two were determined to be dual-tropic viruses while all the remaining isolates were R5-tropic viruses. The earlier set of 41 sequences represented only the ART-naïve participants, as compared to the cohort in this study, which is inclusive of those on ART. Thus, the coreceptor usage in earlier HIV-1C sequences from Botswana was predominately CCR5 (Table S2 in Supplementary Data). We assessed the reliability of the tools in predicting the coreceptor usage of known HIV-1C X4-tropic viruses. Geno2pheno and WebPSSM were both effective at identifying R5-tropic viruses (97.4% and 94.9% accuracy, respectively) (Table S3 in Supplementary Data) but not at identifying X4-tropic viruses (50% accuracy for each tool). The 11/25 charge rule was efficient in correctly identifying both the R5-tropic viruses (97.4%) and X4-tropic viruses (100%) in this dataset. We further expanded our dataset by using extracted (*n* = 94) R5-tropic and (*n* = 8) X4-tropic sequences from Los Alamos HIV sequence database (http://www.hiv.lanl.gov/, acessed on 16 May 2022). The sequence search interface in the HIV sequence database was used with the following terms: (subtype C AND phenotype: NSI/SI AND coreceptor: all CCR5/all CXCR4 AND genotypic region: V3). The tropism of the sequences was phenotypically and genotypically predicted. We assessed the reliability of the same three genotypic tools: Geno2pheno, WebPSSM and 11/25 rule. All three tools effectively identified R5- and X4-tropic viruses (Table S4 in Supplementary Data). The accuracy of geno2pheno, WebPSSM and 11/25 charge rule for R5 -tropic viruses was 98.9%, 96.8% and 100%, respectively. In addition, the accuracy of predicting X4-tropic was also high with 87.5% geno2pheno, 87.5% WebPSSM and 100% for 11/25 rule.

### 3.3. Potential Associations between Viral Tropism and Demographic/Clinical Characteristics of PLWH

We stratified sequences that were classified concordantly by all three tools as either R5 (*n* = 3367) or X4 (*n* = 196) by gender, age group, ART status, viral load and stage of HIV-1 infection. The demographic/clinical characteristics of PLWH statistical analysis excluded the 16 sequences, for which all three tools failed to predict viral tropism. We found no difference in viral tropism between groups stratified by gender or viral load status (*p* > 0.05; Table 3). However, ART status and age were associated with viral tropism. Patients on ART were more likely to harbor X4-tropic viruses (*p* = 0.03; Figure S2 in Supplementary Data). Participants aged 36–45 were more likely to harbor X4-tropic viruses (*p* = 0.006). The viral load status in Table 3 is inclusive of naïve and participants on ART, as viral suppression in our study was defined as virologic suppression with viral load ≤40 copies and virologic unsuppressed with viral load >40 copies. We further categorised viral load status (suppressed and unsuppressed) inclusive of only participants on ART and we did not see any difference in viral tropism (*p*-value = 0.86). The discordant cases showed no significant difference in all clinical backgrounds (*p*-value > 0.05), while the concordant cases showed a significant difference in ART status. There was no difference in viral tropism between the groups in participants on ART and suppressed stratified by viral load, gender, stage of infection and age group.

For 1223 participants, we were able to determine stage of infection as either recent ((<1 year; *n* = 110) or chronic (>1 year; *n* = 1113; Table 3)). There was no statistically significant difference in stage of infection between the groups harboring X4- and R5-tropic viruses (*p* > 0.05).

## 4. Discussion

Botswana HIV-1 prevalence is among the highest in the world at 20.3% in 2018 [20]. Monitoring coreceptor usage is vital, as the emergence of X4-tropic viruses in chronic HIV-1 infections [36] affects the suitability of third-line drugs, such as Maraviroc, for PLWH who experience multi-drug or multi-class resistance. In this study, we utilized seven tools for prediction of HIV-1 tropism in 5602 people living with HIV-1C in Botswana. Due to overlap between some tools, our major analysis focused on three distinct tools: the 11/25 charge rule, Geno2pheno and WebPSSM.

The three tools had a concordance of 64%, consistent with a previous analysis of subtype C sequences [22]. In our study, the highest concordance, 89%, was observed between Geno2pheno and the 11/25 charge rule, while previous studies found the highest concordance between Geno2pheno and WebPSSM [22,37,38]. A potential explanation is that one of the studies conducted in Ethiopia did not use the 11/25 charge rule and used the “clonal” model in Geno2pheno, while we used the “clinical” model [22], as recommended by the European Consensus Group on clinical management of HIV-1 tropism testing [33]. Geno2pheno and WebPSSM had the lowest concordance among all the tools used in our study. According to Riemenschneider et al. [39], WebPSSMsinsiC is less specific in predicting HIV-1C tropism when compared to Geno2pheno, consistent with our finding that the combination of the two tools may give a discordant result. This is explained, at least partially, by differential ways of dealing with insertions, deletions and/or ambiguous positions by each algorithm [22]. We realize that more analysis might be needed to train WebPSSM, and Geno2pheno tools on predicting the X4-tropic viruses in HIV-1C. It is also likely that the three tools might be more effective if they could distinguish between dual-tropic viruses and pure X4-tropic viruses as phenotypic tests do. Therefore, we suggest that the combined usage of tools could be beneficial and the discrepancy across the tools would indicate the extent of uncertainty in estimating viral tropism. We suggest that future work should include analysis using both phenotypic tests and genotypic tests, which was beyond the scope of this study. This study cannot provide clinicians with a definite answer on the antiretroviral drugs to prescribe for discordant cases. Nonetheless, we suggest that genotyping resistance testing must be conducted to determine the drug mutations present and assign the appropriate ART according to Botswana ART guidelines. Phenotyping testing is also recommended. Other clinical parameters should also be taken into account, consistent with previous studies’ suggestion of administering alternative drug regimens before considering Maraviroc [36]. If the participant has gone through all possible options of combination ART and is in need of salvage therapy, we recommend the use of Maraviroc alongside other salvage therapy drugs in Botswana. The participant should continue to be closely monitored. However, we stress that these recommendations should not be followed without guidance from a clinical professional. We hope the results from this study inspire the development of more sensitive subtype C genotypic tools in the future that are readily available and accessible.

Most tools classify dual-tropic viruses as X4-tropic viruses, and we followed this principle in our study. The dual-tropic viruses may possibly represent an intermediate stage in the evolution of the coreceptor [35]. Some studies show that the CCR5 antagonist Maraviroc is active against dual-tropic viruses, which supports its use in dual-tropic viruses [40,41]. However further investigations are needed to validate these findings.

The concordant frequency of X4-tropic viruses (3.5%) by the three tools was lower than the previous rate of 14.5% reported in Botswana based on phenotypic assays [11] and the rate of 33% estimated using Geno2pheno [3]. However, these studies were performed among people with advanced disease (treatment-naïve with low CD4^+^ T-cell counts) and those who were failing treatment, respectively. In contrast, our study participants represent the general population at different stages of disease, including recently infected individuals. The two studies [3,11] had small sample sizes (<200 participants per study), while our study comprised over 5000 participants, constituting the largest study undertaken thus far in Botswana.

There was a significant difference in ART status between groups (*p* = 0.03), indicating that PLWH on ART were more likely to harbor X4-tropic viruses than ART-naïve PLWH. The emergence of X4-tropic viruses in PLWH on ART is a phenomenon that is not fully understood. It has been suggested that treatment may reduce the expression of R5-tropic viruses in association with a reduced immune response, triggering the selection for X4-tropic viruses [6]. Even though treatment might have an impact on tropism, there was no difference in HIV-1 RNA levels between the two groups. This could mean a switch from CCR5 to CXCR4 coreceptors in HIV-1C infection is limited or absent, unlike subtypes B and D [17]. It is possible that X4-tropic viruses could be present in acute infection. Alternatively, low CD4^+^ T-cell count before treatment initiation might influence the emergence of X4-tropic viruses [6]. There was a significant difference in tropism by age groups, indicating that the 36-45-year-old age group was more likely to harbor X4-tropic viruses. It has been shown that this age group has a high coverage of receiving ART treatment but lower viral suppression compared to other age groups [24], which may have led to this observed pattern. Furthermore, we found no difference in coreceptor usage in the early and chronic stages of infection (< or >1 year). In contrast, an African meta-analysis of coreceptor tropism found a 3-5-fold increase in X4-tropic viruses during chronic infection [36]. The disparity is likely due to the difference in the definition of chronic stage, as HIV-1 infections ≥5 years were classified as chronic in the meta-analysis [36], while we defined chronic infections as >1 year, based on the data available. Therefore, the majority of chronic infections in our study were more recent than those in the meta-analysis. Nonetheless, the frequency of recent infections among R5-tropic viruses was twice as high as the frequency of recent infections among X4-tropic viruses, but the difference was not statistically significant, possibly because of the small number of X4-tropic viruses among recent infections. In our study, the HIV-1C tropism trends are analysed from a larger dataset in Botswana of more than 5000 sequences over a period of 5 years (2013–2018). Our current study captures a more accurate current picture of the HIV-1C tropism trends in the Botswana epidemic when compared to the Matume et al. [36] study. Their study only analysed 1401 sequences from Botswana over a wider time period, inclusive of previous Botswana cohorts from a wider timeline highlighted by [3,11,13]. The Matume et al. [36] study used HIV-1C sequences from different countries in Africa where the epidemic has been managed differently, particularly pertaining access to antiretroviral therapy. As a result, the trends in HIV-1 coreceptor use in Matume et al. [36] could have been impacted by this. Botswana has been improving the inclusivity of its treatment guidelines over the years [18]. This shows a novel finding in our study of the trends on tropism with low X4-tropic viruses and the evolution of the virus of the current epidemic in the era of universal treatment where Botswana has exceeded the UNAIDS of 95-95-95 overall target by achieving 88% viral suppression [21].

Several study limitations should be noted. First, a subset of participants was missing CD4^+^ T-cell count data, which is important in helping to better predict tropism; therefore, it was not included in the final analysis. However, most of the tools successfully determines tropism despite the absence of CD4^+^ T-cell count data. Second, we utilized a single consensus HIV sequence per individual; therefore, we cannot exclude the possibility of X4-tropic viruses as minority variants and comment on the tropism changes. Analysis of minor viral variants was beyond the scope of this study. We did not evaluate viral tropism according to time on ART as this was beyond the scope and design of this study and the original study [25,26].

## 5. Conclusions

In conclusion, we demonstrated that R5-tropic viruses are predominant in HIV-1C among the general population in Botswana. Consequently, the use of Maraviroc in PLWH who experience multi-drug and/or multiclass resistance and have limited treatment options should be feasible in Botswana with tropism testing. HIV-1 tropism should continue to be monitored using genotypic tools, particularly for PLWH with limited treatment options, and we suggest that the tools for coreceptor prediction should be used in combination. Further analysis is needed to understand the mechanism of impact of ART in X4-tropic viruses.

## Figures and Tables

**Table 1:** Prediction of coreceptor usage using 7 genotypic tools, *n* = 5602 HIV-1C sequences.

Tools	R5-Tropic Viruses	X4-Tropic Viruses	Failed *
Geno2pheno	4933 (88.3%)	620 (11.1%)	49
WebPSSM	3459 (61.9%)	2128 (38.1%)	15
11/25 Rule	5206 (93.2%)	380 (6.8%)	0
Random Forest	4866 (94.2%)	299 (5.8%)	437
Support Vector Machine	4572 (88.5%)	593 (11.5%)	437
Decision Tree	4913 (95.1%)	252 (4.9%)	437
Neutral Network	4506 (87.2%)	659 (12.8%)	437

* V3 loop sequences shorter than the 35 amino acids; some genotypic tools could not determine tropism. WebPSSM: Web Position-Specific Score Matrices.

**Table 2:** Concordance of the three tools.

Tools	R5	X4	Unpredicted Tropism	Total	Concordance
THREE Tools	3367	196	16	3579	63.9%
Geno2pheno *-WebPSSM	3322	483	15	3820	68.2%
Geno2pheno *-11/25 Rule	4750	192	0	4942	88.5%
WebPSSM-11/25 Rule	3445	366	0	3811	68.2%

* Geno2pheno tool used with a false-positive rate cutoff of 10%, as per user manual instructions.

**Table 3:** Characteristics of the PLWH with congruent tropism predicted by the three tools.

Characteristics	*n* = 3563	R5-Tropic Viruses	X4-Tropic Viruses	*p*-Value
sex	*n* = 3454			
Female	2419	2277 (69.7%)	142 (75.1%)	0.14
Male	1035	988 (30.3%)	47 (24.9%)	
Age groups	*n* = 3454			
16–25 years old	287	273 (8.4%)	14 (7.4%)	0.006
26–35 years old	883	845 (25.9%)	38 (20.1%)	
36–45 years old	1211	1121 (34.3%)	90 (47.6%)	
46–55 years old	713	679 (20.8%)	34 (18.0%)	
56–66 years old	360	347 (10.6%)	13 (6.9%)	
art status	*n* = 3433			
ART Naive	870	835 (25.7%)	35 (18.5%)	0.03
On ART	2563	2409 (74.3%)	154 (81.5%)	
viral load Status (ALL)	*n* = 3291			
Virologically Suppressed *	2111	1992 (64.0%)	119 (67.2%)	0.42
Virologically Unsuppressed *	1180	1122 (36.0%)	58 (32.8%)	
stage of infection	*n* = 1223			
Chronic	1113	1049 (90.7%)	64 (95.5%)	0.27
Recent	110	107 (9.3%)	3 (4.5%)	

* Virologic status was defined as virologic suppression with viral load ≤40 copies and virologic unsuppressed with viral load > 40 copies.

## Data Availability

HIV-1 sequences, basic demographic and clinical data are available when requested from the PANGEA consortium (https://www.pangea-hiv.org/, accessed on 25 January 2023). The BCPP data and protocols are publicly available (https://data.cdc.gov/Global-Health/Botswana-Combination-Prevention-Project-BCPP-Publi/qcw5-4m9q, accessed on 25 January 2023).

## Supplementary Materials

The following supporting information can be downloaded at https://www.mdpi.com/article/10.3390/v15020403/s1, Table S1: X4-Tropic Viruses distribution across the three tools; Table S2: Prevalence of R5 and X4-tropic viruses among sampling years; Table S3: Prediction of coreceptor usage using early Botswana HIV-1C sequences; Table S4: Prediction of coreceptor usage using previously phenotypically determined HIV-1C sequences tropism from Los Alamos Database; Figure S1: The distribution of X4-tropic viruses in all the three tools; geno2pheno, WebPSSM and 11/25 rule; Figure S2: ART status between the R5-tropic viruses and X4-tropic viruses represented by percentages and χ^2^ test was used to calculate the *p*-value = 0.03.

## Appendix A

PANGEA Consortium:

Lucie Abeler-Dörner ^1^, Helen Ayles ^2^, David Bonsall ^1^, Rory Bowden ^3^, Vincent Calvez ^4^, Sarah Fidler ^5^, Christophe Fraser ^1^, Kate Grabowski ^6,7^, Tanya Golubchik ^1^, Richard Hayes ^8^, Joshua Herbeck ^9^, Joseph Kagaayi ^7^, Pontiano Kaleebu ^10^, Jairam Lingappa ^9^, Deenan Pillay ^11,12^, Thomas Quinn ^6,13^, Andrew Rambaut ^14^, Oliver Ratmann ^15,16^, Janet Seeley ^8^, Deogratius Ssemwanga ^10^, Frank Tanser ^12^, Maria Wawer ^7,17^, Myron Cohen^18^, Tulio D’Oliveira ^19^, Ann Dennis ^18^, Dan Frampton ^20^, Anne Hoppe ^20^, Paul Kellam ^21^, Cissy Kityo ^22^, Andrew Leigh-Brown ^14^, Nick Paton ^23^

^1^ Oxford Big Data Institute, Li Ka Shing Centre for Health Information and Discovery, Nuffield Department of Medicine, University of Oxford, Oxford, UK; ^2^ Zambart Project, Lusaka, Zambia; ^3^ Oxford Genomics Centre, The Wellcome Centre for Human Genetics, University of Oxford, Oxford, UK; ^4^ Ecole Normale Supérieure de Lyon, Lyon, France; ^5^ Department of Medicine, Imperial College London, London, UK; ^6^ Department of Medicine, Johns Hopkins School of Medicine, Baltimore, MD, USA; ^7^ Rakai Health Sciences Program, Entebbe, Uganda; ^8^ London School of Hygiene & Tropical Medicine, London, UK; ^9^ Department of Global Health, University of Washington, Seattle, WA, USA; ^10^ MRC/UVRI, Entebbe, Uganda; ^11^ Division of Infection and Immunity, University College London, London, UK; ^12^ Africa Health Research Institute, Durban, South Africa; ^13^ Division of Intramural Research, National Institute of Allergy and Infectious Diseases, NIH, Bethesda, MD, USA; ^14^ School of Biological Sciences, University of Edinburgh, Edinburgh, UK; ^15^ Department of Mathematics, Imperial College London, London, UK; ^16^ Department of Infectious Disease Epidemiology, School of Public Health, Imperial College London, London, UK; ^17^ Department of Epidemiology, Johns Hopkins Bloomberg School of Public Health, Baltimore, MD, USA; ^18^ Department of Medicine, University of North Carolina, Chapel Hill, NC, USA; ^19^ College of Health Sciences, University of KwaZulu-Natal, Durban, South Africa; ^20^ Division of Infection and Immunity, University College London, London, UK; ^21^ Department of Medicine, Imperial College London, London, UK; ^22^ Joint Clinical Research Centre, Kampala, Uganda; ^23^ Medical Research Council, London, UK.
