# Supplementary material for: Prediction of Coreceptor Tropism in HIV-1 Subtype C in Botswana
Source: Viruses. 2023 Jan 31;15(2):403. doi: 10.3390/v15020403 (PMC9963705; doi:10.3390/v15020403)
Supplement: Supplementary file 1 [file viruses-15-00403-s001.zip › viruses-2165644-supplementary.pdf]

Table S1. X4- Tropic Viruses distribution across the three tools .

Tools	X4- tropic viruses	X4-tropic viruses (%)
Geno2pheno	620	11.1
WebPSSM	2127	38.0
11/25 RULE	380	6.8
Total	5602	

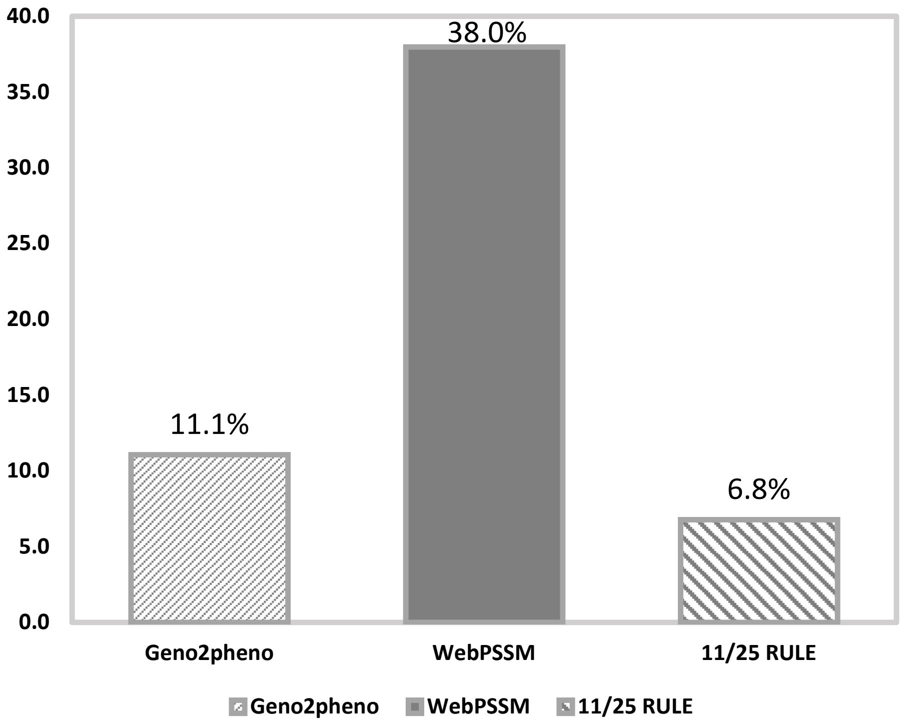

Figure S1. The distribution of X4-tropic viruses in all the three tools; geno2pheno, WebPSSM and 11/25 rule. WebPSSM: Web Position-Specific Score Matrices.

Table S2. Prevalence of R5 and X4- tropic viruses among sampling years.

N = 3023	2013	2014	2015	2016	2017	2018	Total
**R5-tropic viruses**	46 (92%)	422 (94.8%)	1578 (94.4%)	673 (94.3%)	100 (93.5%)	35 (97.2%)	2854
**X4-tropic viruses**	4 (8%)	23 (5.2%)	93 (5.6%)	41 (5.7%)	7 (6.5%)	1 (2.8%)	169

Table S3. Prediction of coreceptor usage using early Botswana HIV-1C sequences.

	Geno2pheno	WebPSSM	11/25 Rule
R5-tropic viruses correctly predicted (n=39)	38 (97.4%)	37 (94.9%)	38 (97.4%)
X4-tropic viruses correctly predicted (n=2)	1 (50%)	1 (50%)	2(100%)

Table S4. Prediction of coreceptor usage using previously phenotypically determined HIV-1C sequences tropism from Los Alamos Database.

	Geno2pheno	WebPSSM	11/25 Rule
R5-tropic viruses correctly predicted (n=94)	93 (98.9%)	94 (96.8%)	94 (100%)
X4-tropic viruses correctly predicted (n=8)	7(87.5%)	7 (87.5%)	8(100%)

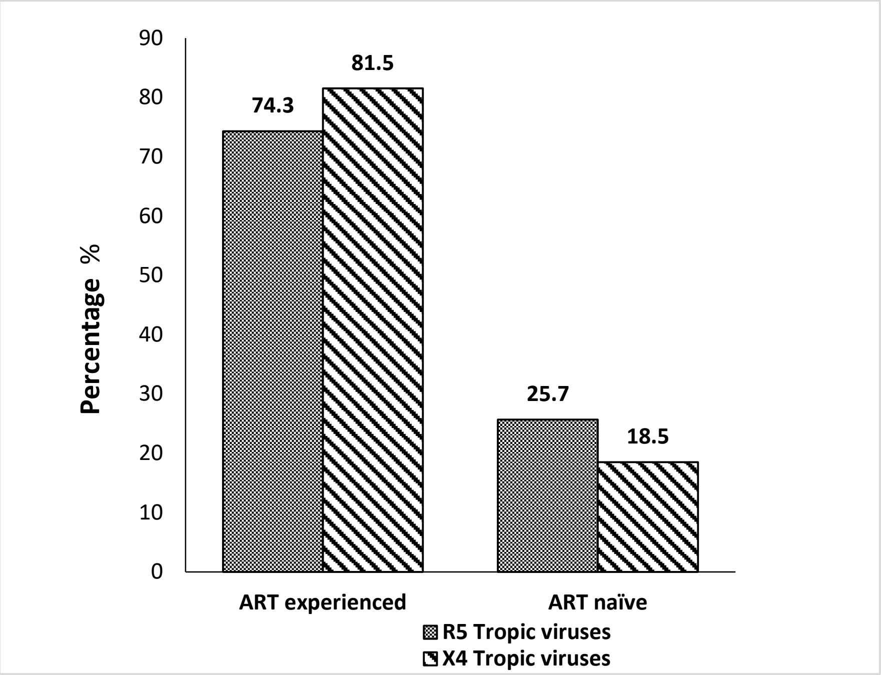

Figure S2. ART status between the R5-tropic viruses and X4-tropic viruses represented by percentages and χ^2 test was used to calculate the p-value =0.03.
